# Small-signal modulation characteristics of a polariton laser

**DOI:** 10.1038/srep11915

**Published:** 2015-07-08

**Authors:** Md Zunaid Baten, Thomas Frost, Ivan Iorsh, Saniya Deshpande, Alexey Kavokin, Pallab Bhattacharya

**Affiliations:** 1 Center for Photonics and Multiscale Nanomaterials, Department of Electrical Engineering and Computer Science, University of Michigan, 1301 Beal Avenue, Ann Arbor, MI 48109, USA; 2 National Research University for Information Technology, Mechanics and Optics (ITMO), St. Petersburg 197101, Russia; 3 Russian Quantum Center, Novaya 100, 143025 Skolkovo, Moscow Region, Russia; 4 School of Physics and Astronomy, University of Southampton, SO17 1NJ Southampton, United Kingdom

## Abstract

Use of large bandgap materials together with electrical injection makes the polariton laser an attractive low-power coherent light source for medical and biomedical applications or short distance plastic fiber communication at short wavelengths (violet and ultra-violet), where a conventional laser is difficult to realize. The dynamic properties of a polariton laser have not been investigated experimentally. We have measured, for the first time, the small signal modulation characteristics of a GaN-based electrically pumped polariton laser operating at room temperature. A maximum −3 dB modulation bandwidth of 1.18 GHz is measured. The experimental results have been analyzed with a theoretical model based on the Boltzmann kinetic equations and the agreement is very good. We have also investigated frequency chirping during such modulation. Gain compression phenomenon in a polariton laser is interpreted and a value is obtained for the gain compression factor.

The proposal for generating coherent light by spontaneous emission from a coherent, macroscopic and degenerate exciton-polariton condensate in a microcavity^1^ was followed by experimental demonstration of such emission at cryogenic^2^,^3^,^4^,^5^,^6^,^7^,^8^,^9^ and higher temperatures, including room temperature^10^,^11^,^12^,^13^,^14^,^15^,^16^,^17^,^18^, in a variety of material systems. The device is commonly termed a polariton laser. Lack of the need for population inversion, as in a conventional laser, inherently enables coherent emission with thresholds that are generally 2–3 orders of magnitude lower than those measured for photon lasing in the same device^2^,^5^,^7^,^13^,^16^,^18^,^19^. The characterization of optically or electrically pumped polariton lasers made with a variety of material systems has enabled a detailed study of the underlying physical processes such as polariton scattering and Bose-Einstein condensation^20^,^21^,^22^,^23^, spontaneous symmetry breaking^24^ and superfluidity^25^ in the condensate. However, all the results hitherto reported have been obtained under steady state conditions with continuous wave electrical biasing or optical excitation. The small-signal modulation bandwidth of a conventional semiconductor laser is intrinsically limited by gain compression and related hot carrier effects^26^. Since polariton lasers operate at much lower injection levels, it is expected that the intrinsic modulation bandwidth would not be similarly affected^27^. Other related effects such as chirp and self-pulsation should be small or non-existent. Dynamic characterization would also elucidate the similarities, or lack thereof, in terms of key lasing parameters such as differential gain and damping factor and the physical processes underlying them in a polariton laser.

We have studied experimentally the bulk GaN microcavity diode schematically shown in Fig. 1(a). Molecular beam epitaxial growth and fabrication of the device are described in Supplementary information. All measurements in the present study have been made at room temperature (see Methods). The measured lower polariton (LP) dispersion is depicted in Fig. 1(b). Analysis of the data with the coupled oscillator model^28^ allows us to determine the cavity-to-exciton detuning *δ* = −10 meV and the Rabi splitting Ω = 33 meV. The variation of integrated intensity with current is depicted in Fig. 2(a). The inset to this figure shows the output spectrum for 220 A/cm^2^ forward current density. A nonlinear threshold is observed in the characteristics at 190 A/cm^2^, which is very similar to the value previously reported for a bulk GaN microcavity^18^. Furthermore, as previously observed, the slope of the output characteristics in the pre-threshold region is sublinear (~0.8), which we believe is due to non-radiative recombination and carrier leakage from the active region. The LP density at threshold is calculated to be 2.28 × 10^16^ cm^−3^ using the relation 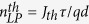 where the exciton lifetime of *τ* = 0.57 ns, as measured in an identical sample, is used. This threshold density is significantly smaller than the transparency density (~3 × 10^18^ cm^−3^)^29^ and the Mott density (1 − 2 × 10^19^ cm^−3^)^10^ reported for GaN. The injection current was further increased, using pulsed mode bias beyond 1.5 kA/cm^2^, and a second non-linear threshold is observed at 46 kA/cm^2^ (Fig. 2(b)). This non-linearity, which is accompanied by a three-orders-of-magnitude increase in the output power compared to that in the polariton lasing regime, is due to conventional photon lasing. The measured variation of the linewidth of the LP emission with injection is plotted in Fig. 2(c). The minimum linewidth of 1.95 meV corresponds to a LP coherence time of 2.12 ps. Beyond the minimum, the linewidth increases again due to exciton-exciton interactions. Also plotted in Fig. 2(c) is the measured blueshift of the LP emission peak, caused by polariton-polariton and polariton-exciton interactions^30^. To confirm the dynamic condensation of polaritons in the microcavity, the LP population distribution in k-space both below and above threshold was measured (see Methods). The polariton occupation in k-space at different injection levels is shown in Fig. 2(d). It is evident that the LP population distribution is random below threshold, which transforms to a peaked occupancy at 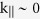 above threshold indicating the formation of a degenerate polariton condensate.

To investigate and analyze the dynamic characteristics of the polariton laser, we have measured the small signal modulation response of the device. The modulation response at various injection levels is shown in Fig. 3(a). A −3 dB modulation bandwidth of 1.18 GHz was measured at an injection current density of 5.4 J_th_ and the resonance frequency, *f*_*r*_, at this injection level is 0.9 GHz. The solid curves in Fig. 3(a) represent the modulation response calculated in accordance with the transfer function described in the Methods section. Emission is stimulated in a conventional photon laser, whereas in the polariton laser polariton-polariton scattering is stimulated once the occupation is unity. The separation of stimulation and emission in a polariton laser leads to coherent emission without the requirement for population inversion. Iorsh *et al.*^27^ have theoretically investigated the small-signal modulation behavior of III-nitride electrically pumped polariton lasers. According to their theory, the resonance frequency of the modulation response, *ω*_*R*_(pol), in the framework of the Boltzmann kinetic model (see Methods for details), is given by:


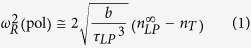


where 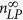 is the steady-state polarit*o*n density at 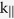 ∼  0,*b* is the polariton-polariton scattering rate beyond threshold and *τ*_*LP*_ is the LP radiative lifetime. In equation (1), we have added the term *n*_*T*_ as compared to the expression in^27^. This term accounts for the fraction of the polariton states which have been trapped in localized states and dislocations and do not contribute to the laser kinetics. Of course, the right hand-side of equation (1) should always be positive within the limits of validity of this model, i.e., conditions above threshold. Equation (1) is similar in nature to that for a photon laser, where 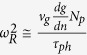. Here *v*_*g*_, *dg*/*dn*, *N*_*p*_ and *τ*_*ph*_ are the photon group velocity, differential gain, photon density in the cavity and cavity photon lifetime, respectively. Gain and differential gain are related to stimulated emission in a photon laser. Polariton lasing arises from stimulated polariton scattering with excitons, free carriers or phonons. This results in gain, which may be described by the negative imaginary part of the refractive index. Gergel’ *et al.* have described gain induced by exciton Bose condensation and have derived the complex dielectric function explicitly^31^. Phenomenologically, therefore, a differential gain in a polariton laser may be expressed as,


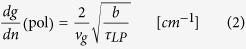


The important aspect of this equation is that the differential gain is large for a large scattering rate *b* and small *τ*_*LP*_, i.e. a large coupling strength and a negative detuning. It may be noted that the small-signal modulation response depicted in Fig. 3(a) is damped. The damping factor γ_d_ can be derived from analysis of the modulation response. For large resonance frequency, the damping factor in a polariton laser is given by 
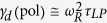
27. In a well-designed photon laser damping is generally a result of gain compression due to the accumulation of hot carriers in the active region and subsequent spectral and spatial hole burning. In a polariton laser, such damping can be caused by saturation of the oscillator strength by phase space filling^32^ at high injection levels and the decrease of the Rabi oscillation frequency and the resultant exciton-photon coupling. Hence the damping factor can be expressed as,


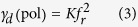


where *K* = 4*π*^2^*τ*_*LP*_. Including gain compression, the *K*-factor of a polariton laser may phenomenologically be expressed as,


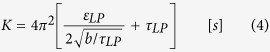


where the gain compression factor *ε*_*LP*_ is dimensionless. To obtain the numerical dependence of resonant frequency on the polariton occupation number and the value of *ε*_*LP*_, we must evaluate the exciton-exciton scattering rate. This can be extracted from the simple three level rate equations of the polariton laser (details discussed in Methods). The value of *b* is obtained by setting the threshold current density to the experimentally observed value of 190 A/cm^2^. This gives us *b* = 0.7 × 10^−12^ ps^−1^, which is very close to the value obtained in the simulations^27^. Figure 3(b) shows a plot of the measured resonance frequency versus the square root of the polariton occupation in the ground state. The solid line represents analysis of the data in accordance with equation (1). The value of *n*_*T*_ = 1.1 was obtained from the fitting of the experimental data to equation (1). The damping factor *γ*_*d*_ is plotted against the square of the resonance frequency in Fig. 3(c). From the slope of this plot, a value of *K* = 5.02 ns is derived. The intrinsic −3 dB modulation bandwidth of the polariton laser, given by *f*_−3*dB*_ = 2^3/2 ^ *π*/*K*, is determined to be 1.77 GHz. The differential gain is calculated to be 1.89 × 10^−4^ cm^−1^ from equation (2) using the values of *b* and *τ*_*LP*_ quoted above. Finally, the gain compression factor is derived from equation (4) to be *ε*_*LP*_ = 2.78 × 10^−4^ . In comparison, the differential gain and gain compression factor of GaN-based red-emitting InGaN/GaN quantum dot lasers are reported to be 5.3 × 10^−17^ cm^2^ and 2.87 × 10^−17^ cm^3^, respectively^33^. The *K-*factor reported for a similar green emitting quantum dot laser is 1.24 ns and the corresponding gain compression factor and differential gain are 1.22 × 10^−17^ cm^3^ and 3 × 10^−17^ cm^3^ respectively^34^.

Frequency chirping in a semiconductor photon laser is generally a result of periodic modulation of the refractive index of the gain medium due to injection of carriers. Chirp is small in lasers having a large differential gain, such as quantum dot lasers^35^, in which the carrier injection levels are small. Chirp manifests itself in the broadening of the emission spectra. With an injection carrier density of ~10^15^ cm^−3^ in the polariton laser the estimated shift of the emission peak, Δλ ~ 0.056 Å. The data shown in Fig. 3(d) indicate that the expected near-zero chirp is within the measurement error limits. This is advantageous for possible modulated polariton laser applications.

In conclusion, we have investigated the small-signal electrical modulation characteristics of a polariton laser for the first time. The measurements were conducted at room temperature. The variation of the resonance frequency, derived from the measured modulation response, with polariton occupation in the ground state was analyzed with a rate equation model from which the polariton-polariton scattering rate is derived. The linewidth of coherent emission beyond threshold exhibits negligible broadening with high frequency modulation, which indicates that chirp is negligible. While the modulation bandwidth and damping are derived from the measured modulation response, differential gain and gain compression in a polariton laser are phenomenologically defined, calculated and interpreted in terms of lasing parameters.

## Methods

### Photoluminescence measurements

Photoluminescence measurements were performed on a bulk GaN-on-sapphire sample at 25 and 300 K using a 325 nm He-Cd laser. The quality factor of the microcavity was estimated from microphotoluminescence measurements made at 300 K. The experimental details are provided in the Supplementary information.

### Polariton dispersion and light-current characteristics

The polariton dispersion characteristics were determined from angle-resolved electroluminescence measurements (see Supplementary information). The lower polariton (LP) transition is clearly seen at all angles, with its spectral position approaching the X_A_ resonance at large angles. No other transitions, including the upper polariton (UP), were discernible in the spectra.

The light-current (L-I) characteristics of the device were determined by recording the electroluminescence in the direction normal to the distributed Bragg reflector (DBR) mirrors (zero angle) as a function of continuous wave injection current. Two methods were used to record the L-I characteristics. In the first the integrated intensity of the polariton emission was plotted as a function of forward bias current. In the second, the output power was directly measured with a power meter. Both techniques yielded identical trends in the characteristics.

### Polariton occupation in k-state

The polariton occupation in k-space at different injection levels was measured by angle-resolved electroluminescence, using a digital readout angular mount having a precision of 0.1°. Below threshold, the number of polaritons per k-state is estimated from the LP electroluminescence integrated intensity by taking into account the radiative lifetime. At and above threshold, the occupation is calculated from the output power measured with a power meter.

The polariton occupation number per *k*_||_ state is calculated by using the formula, 
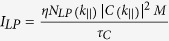
 , where *η* is the collection efficiency, 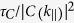 is the radiative lifetime of LPs, *M* is the number of transverse states included in the detection cone, and |*C(k*_*||*_)|^2^ is the photon fraction at a wave vector 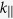 . The number of states within the detection cone is given by *M* = *D*^2^/16(*k*_*o*_Δ*θ*)^2^, where *D* is the diameter of the emission spot, *k*_*o*_ = 2*π*/*λ* and Δ*θ* is the detection half angle.

### Transient response and chirp

The experimental setup for the measurement of the transient response of the device consists of the following: GSG probe, bias tee, optical fiber, single photon detector, single photon counter, AC pulse generator, DC source meter, triple grating monochromator, Peltier cooler and temperature controller. The transient response was measured by superimposing a small-signal periodic switching pulse (1–3 mV) on different DC bias voltages set above the polariton lasing threshold. The modulation response was derived by the fast Fourier transform (FFT) of the measured transient response, which was recorded using a monochromator (resolution 0.03 nm), a high-speed single photon detector and a single photon counter. The RC effect of the microcavity diode was taken into account in the modulation response.

In measuring chirp of the polariton laser, we recorded the average broadening of the coherent emission spectra under small signal (2 mV) pulsed bias condition above threshold (1.15 J_th_) for different modulation frequencies.

### Modulation transfer function

The response at different injection levels is analyzed by the response function:


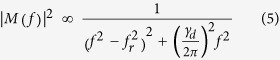


where *γ*_*d*_ is the damping factor and *f*_*r*_ is the resonance frequency.

### Rate equations

In the steady state the occupation numbers of the polariton ground state 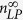 , reservoir occupation number 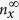 and the number of free carriers in the system 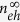 can be obtained from the following Boltzmann kinetic equation set:


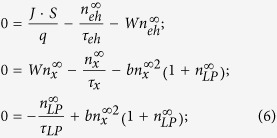


where *J* is the current density, *q* is the electron charge, *S* is the contact surface area equal to 138 μm^2^, *W* is the exciton formation rate equal to 0.01 ps^−1^ (ref. 27), and are *τ*_*eh*_ = 1 ns,*τ*_*x*_ = 0.6 ns, *τ*_*LP*_ = 0.58 ps are the free carrier, exciton and ground state polariton lifetimes.

## Additional Information

**How to cite this article**: Zunaid Baten, M. *et al.* Small-signal modulation characteristics of a polariton laser. *Sci. Rep.* **5**, 11915; doi: 10.1038/srep11915 (2015).

## Supplementary Material

Supplementary Information

## Figures and Tables

**Figure 1:**
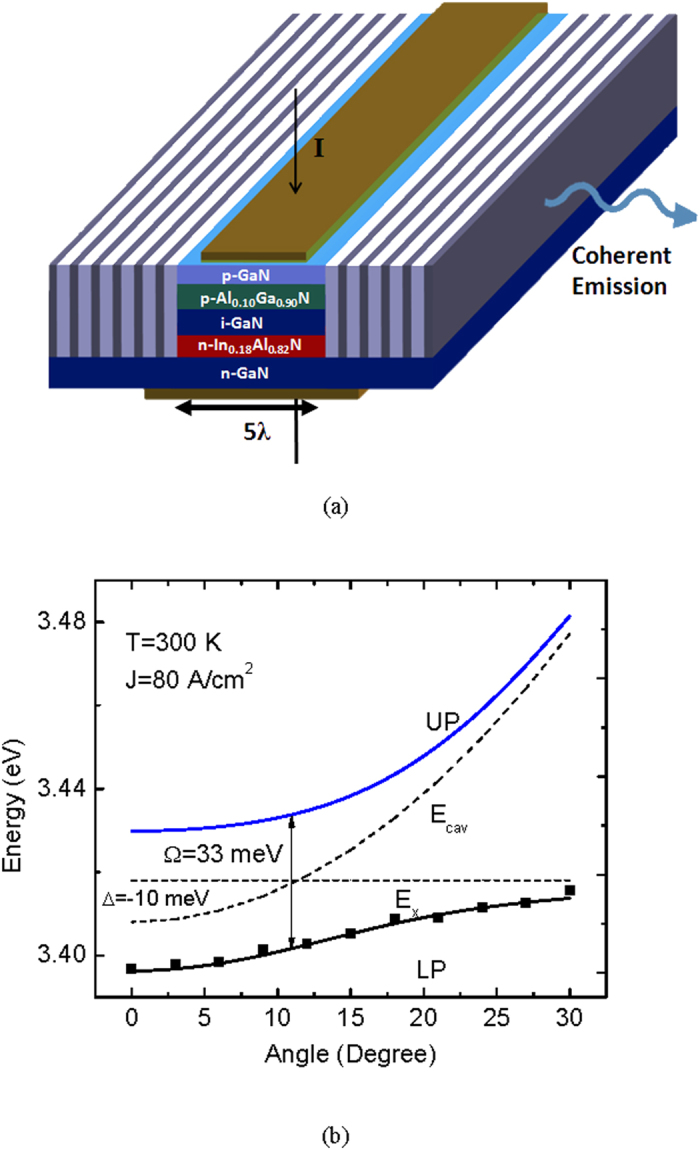
Device schematic and polariton dispersion. **a**, Schematic representation of the bulk GaN-based microcavity diode. The direction of current injection and polariton emission are shown by arrows. **b**, Polariton dispersion characteristics derived from 2 × 2 coupled harmonic oscillator model alongside peak energy positions of the lower polaritons measured by angle-resolved electroluminescence (see Supplementary information).

**Figure 2:**
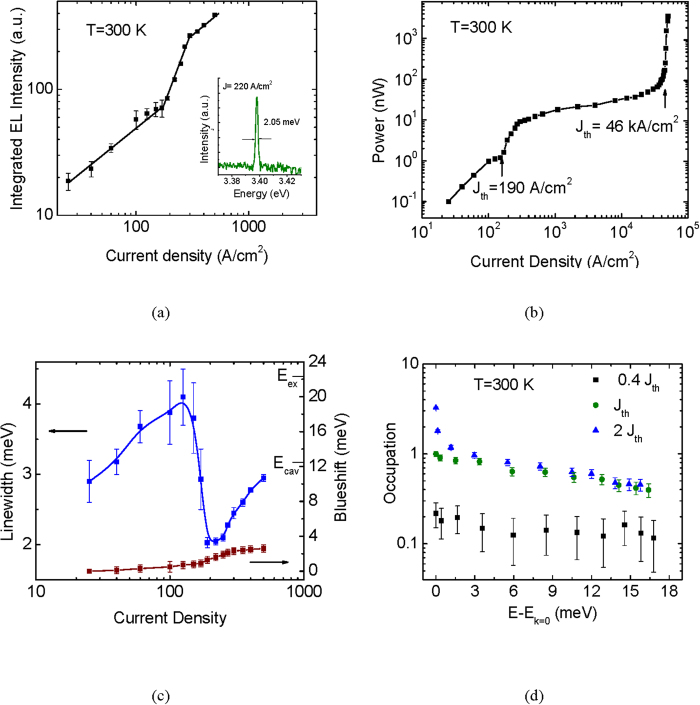
Polariton laser output characteristics in steady state. **a**, Integrated electroluminescence (EL) intensity of the LP emission as a function of injected current density. Inset shows an EL spectra measured at an injection above the lasing threshold. **b**, Two threshold lasing behavior showing the non-linearities due to polariton and photon lasing. The threshold current densities of polariton and photon lasing are indicated by arrows. **c**, Measured LP emission linewidth and blueshift of LP emission peak as a function of injected current density. **d**, LP ground state occupancy for different k_||_ states obtained from angle-resolved EL at three different injection current densities.

**Figure 3:**
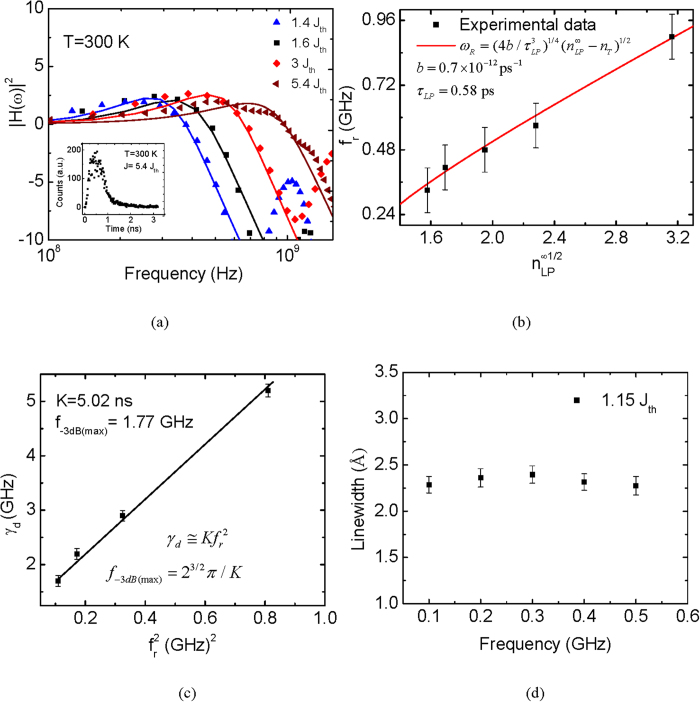
Dynamic characteristics of polariton laser. **a**, Frequency response derived from measured time resolved electroluminescence at different DC injection levels. The solid lines represent the corresponding frequency responses calculated from the modulation transfer function (see Methods). Inset shows the measured transient response of the polariton laser to a high speed switching pulse at J = 5.4J_th_. **b**, Measured resonance frequency as function of 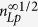 , where 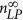 is the steady-state ground state occupancy at 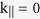. The solid line shows the calculated values from equation (2) for the given scattering rate, *b* and polariton lifetime, *τ*_*LP*_. **c**, Variation of the damping factor as a function of the square of the resonance frequency. **d**, measured linewidth of LP emission peak as a function of the small signal modulating frequency, under a fixed DC bias of 1.15 J_th_.
