# Supplementary material for: Small-signal modulation characteristics of a polariton laser
Source: Sci Rep. 2015 Jul 8;5:11915. doi: 10.1038/srep11915 (PMC4495564; doi:10.1038/srep11915)
Supplement: Supplementary Information [file srep11915-s1.pdf]

Supplementary Information

Small-signal modulation characteristics of a polariton laser

Md Zunaid Baten[1], Thomas Frost[1], Ivan Iorsh[2], Saniya Deshpande[1],
Alexey Kavokin[3,4], and Pallab Bhattacharya[1*]

[1]Center for Photonics and Multiscale Nanomaterials,
Department of Electrical Engineering and Computer Science, University of Michigan,
1301 Beal Avenue, Ann Arbor, MI 48109, USA
[2]National Research University for Information Technology, Mechanics and Optics (ITMO),
St. Petersburg 197101, Russia
[3]Russian Quantum Center, Novaya 100, 143025 Skolkovo, Moscow Region, Russia
[4]School of Physics and Astronomy, University of Southampton, SO17 1NJ Southampton,
United Kingdom
[*]Email: pkb@umich.edu

This file includes:

1. Heterostructure Growth and Device Fabrication

2. GaN Optical Characteristics

3. Cavity Resonance and Angle-resolved Electroluminescence

1. Heterostructure Growth and Device Fabrication

The device heterostructure (schematically shown in Fig. S1(a)) was grown by plasma-assisted molecular beam epitaxy on c-plane GaN-on-sapphire substrate. The 300 nm n (Si) doped $In_{0.18}Al_{0.82}N$ layer, which is lattice matched to GaN, confines the photon and also prevents substrate leakage. The active region consists of 300 nm Si doped n-GaN, 300 nm p (Mg) doped $Al_{0.10}Ga_{0.90}N$ and 110 nm p (Mg) doped GaN. The higher bandgap $Al_{0.10}Ga_{0.90}N$ region acts as an electron blocking layer and also provides photon confinement. Device fabrication is initiated by selective deposition of the Ni-Au p-contact by electron beam evaporation followed by rapid thermal annealing of the contact at 550° C in air for two minutes. Next, a 1 μm x 40 μm cavity region is defined by standard UV lithography. The defined region is selectively etched down to the substrate through the $In_{0.18}Al_{0.82}N$ layer by inductively coupled high density plasma under a Cl_2:Ar environment. The etch rate was calibrated to be ~4.5nm/s. The etching is followed by

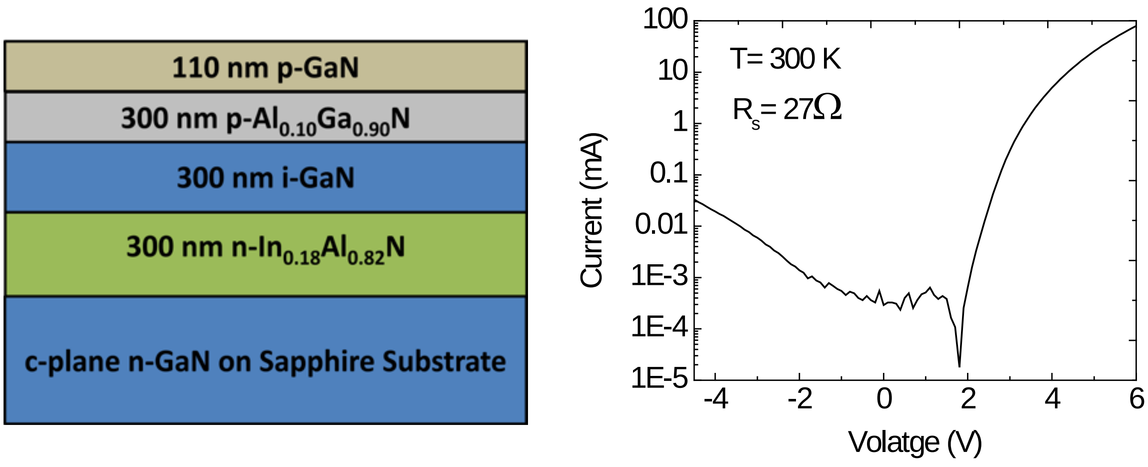

(a)

(b)

Fig. S1: (a) Schematic of the heterostructure; (b) current-voltage characteristics of the device.

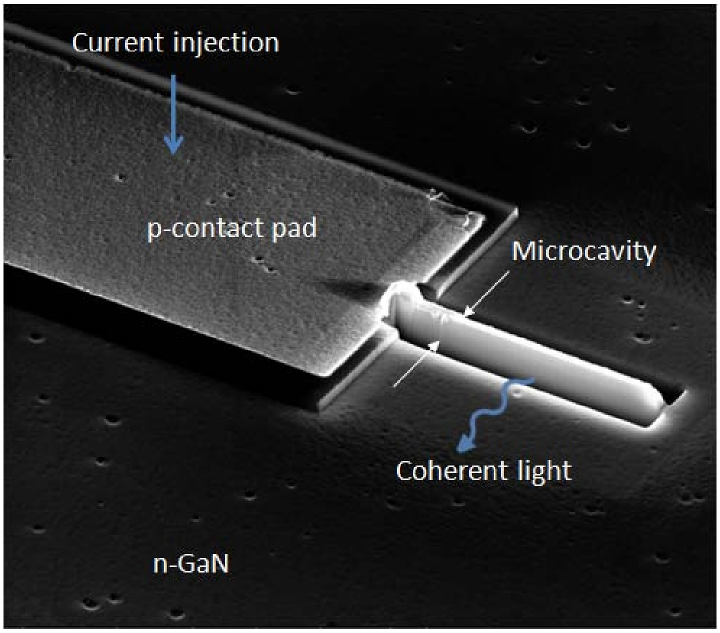

Fig. S2: SEM image of a FIB etched cavity along with the p-contact pad.

selective deposition of Ti/Au by electron beam evaporation onto the n-doped substrate to form the n-contact. Prior to both p- and n-contact deposition, the native oxide was removed by a $HCl:H_2O$ solution.

To form the 690 nm (5λ) x 40 μm cavity, focused ion beam (FIB) etching is done on the previously defined 1 μm x 40 μm region. To ensure optically flat surfaces along the length of the cavity, a final FIB etching is conducted at very low injection. A scanning electron microscope (SEM) image of a 690 nm x 20 μm FIB etched cavity, along with the p-contact pad, is shown in Fig. S2. Finally, using electron beam evaporation, five and six pairs of SiO_2/TiO_2 distributed Bragg reflector (DBR) mirrors are deposited on opposite sides of the FIB etched cavity. The current voltage characteristics of the device at room temperature is shown in Fig. S1(b). The diode is characterized by a turn-on voltage of 4.5V and a series resistance of 27ohms. The leakage current of the diode is less than 1μA and the shunt resistance is ~5.5 MΩ.

2. GaN Optical Characteristics

The excitonic transitions of the active region was studied by photoluminescence (PL) measurements of c-plane GaN-on-sapphire using a 325 nm He-Cd laser. To ensure that the detection geometry of the sample is same as that of the microcavity diode, detection of the photoluminescence was done perpendicular to the c-axis.

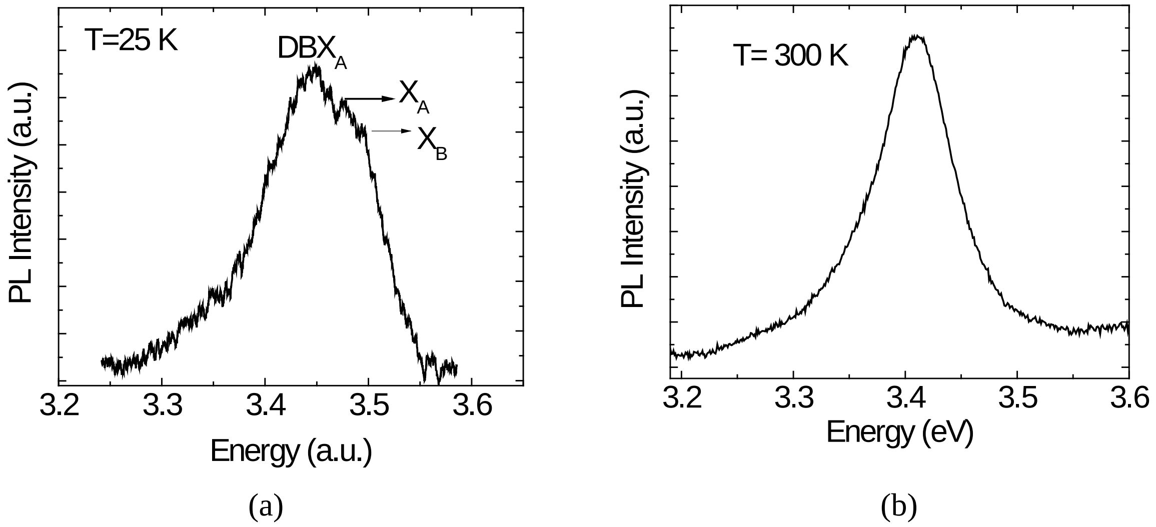

Fig. S2: Measured photoluminescence characteristics of c-plane bulk GaN on sapphire at (a) T=25 and (b) T=300 K.

The PL spectra were recorded using a monochromator having spectral resolution of 0.05 nm. As shown in Fig. S2(a), low temperature PL spectra shows the free excitonic transitions X_A and X_B at 3.475 and 3.491 eV respectively. These energy positions of the excitons are similar to what have been reported before [R1, R2]. The X_A exciton transition was found to be the dominant one with a linewidth of 7.8 meV at T=25 K. The donor bound exciton DBX_A is observed at 3.448 eV, whereas the weaker emission within a lower energy range of 3.36-3.43 eV may be attributed to the surface states. At room temperature, the different exciton peaks merge to show a broad emission having a peak at ~3.418 eV.

3. Cavity Resonance and Angle-resolved Electroluminescence

To measure the quality factor of the microcavity, micro-photoluminescence measurement was performed on the fabricated microcavity diode after DBR deposition. The monochromator used in this study had a spectral resolution of 0.03 nm. Figure S3(a) shows the measured cavity resonance, which has a full-width-half-maximum of 1.8 meV and a peak energy of 3.408 eV. This corresponds to a cavity quality factor of ~1893 and a cavity photon lifetime of 0.366 ps.

Angle resolved electroluminescence (EL) of the polariton laser diode was measured at room temperature using a digital-readout angular mount which has an angular precision of 0.1^{o}. Figure S3(b) shows the angle-resolved EL characteristics measured within the angular range of 0° - 30° under a low forward bias. Here normal direction is considered to be perpendicular to the

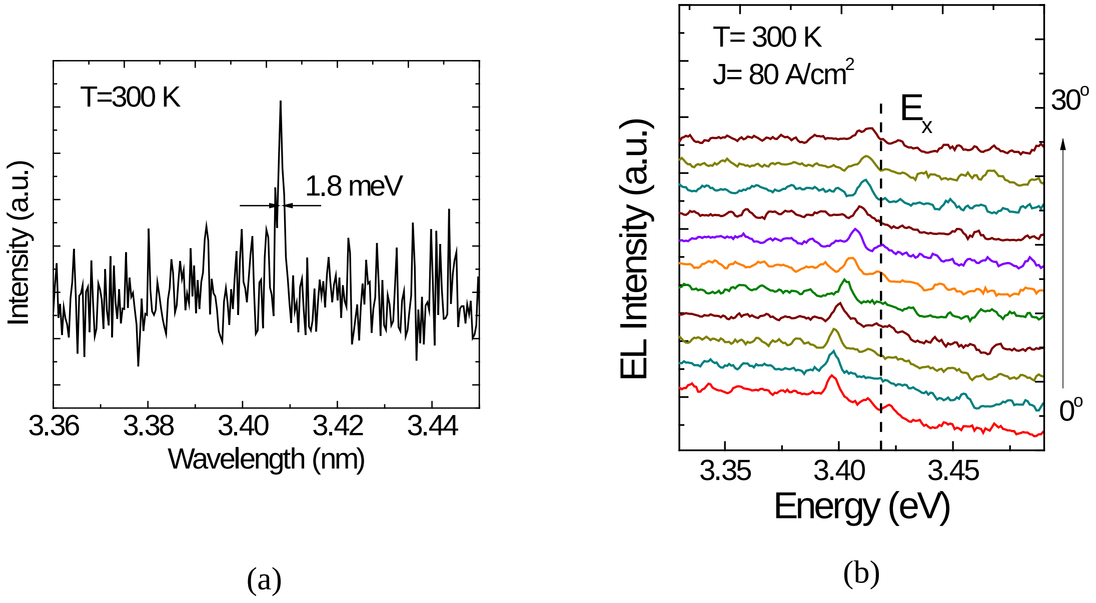

Fig. S3: (a) Measured micro-photoluminescence of the microcavity at room temperature; (b) five point moving average filtered angle-resolved electroluminescence measured from the microcavity diode at room temperature.

DBR mirrors and the measurements are done in the c-plane. Distinct lower polariton (LP) peaks are observed below the exciton energy (E_X = 3.408 eV) at all angles and the LP peaks tend to approach the exciton energy at higher angles. The corresponding polariton dispersion characteristics, which is calculated using 2x2 coupled harmonic model, are shown in Fig. 1(b) of the main text.

References:

[R1] Sellers, I. R. et al. Polariton emission and reflectivity in GaN microcavities as a function of angle and temperature. Phys. Rev. B 74, 193308 (2006).

[R2] Bhattacharya, P. et al. Room temperature electrically injected polariton laser. Phys. Rev. Lett. 112, 236802 (2014).
